# TRPM2 Oxidation Activates Two Distinct Potassium Channels in Melanoma Cells through Intracellular Calcium Increase

**DOI:** 10.3390/ijms22168359

**Published:** 2021-08-04

**Authors:** Loretta Ferrera, Raffaella Barbieri, Cristiana Picco, Paolo Zuccolini, Alessia Remigante, Sara Bertelli, Maria Rita Fumagalli, Giovanni Zifarelli, Caterina A. M. La Porta, Paola Gavazzo, Michael Pusch

**Affiliations:** 1 Biophysics Institute, National Research Council, 16149 Genova, Italy; Loretta.Ferrera@unige.it (L.F.); raffaella.barbieri@ibf.cnr.it (R.B.); cristiana.picco@ibf.cnr.it (C.P.); paolo.zuccolini@ibf.cnr.it (P.Z.); alessia.remigante@ibf.cnr.it (A.R.); sara.bertelli@ibf.cnr.it (S.B.); mariarita.fumagalli@gmail.com (M.R.F.); giovanni.zifarelli@ibf.cnr.it (G.Z.); caterina.laporta@unimi.it (C.A.M.L.P.); paola.gavazzo@ibf.cnr.it (P.G.); 2 U.O.C. Genetica Medica, Istituto di Ricovero e Cura a Carattere Scientifico (IRCCS) Istituto Giannina Gaslini, 16147 Genoa, Italy; 3 Department of Chemical, Biological, Pharmaceutical and Environmental Sciences, University of Messina, 98166 Messina, Italy; 4 Center for Complexity and Biosystems, Department of Environmental Science and Policy, University of Milan, 20133 Milano, Italy

**Keywords:** melanoma, oxidative stress, potassium channels, intracellular calcium, TRP channels

## Abstract

Tumor microenvironments are often characterized by an increase in oxidative stress levels. We studied the response to oxidative stimulation in human primary (IGR39) or metastatic (IGR37) cell lines obtained from the same patient, performing patch-clamp recordings, intracellular calcium ([Ca^2+^]_i_) imaging, and RT-qPCR gene expression analysis. In IGR39 cells, chloramine-T (Chl-T) activated large K^+^ currents (KROS) that were partially sensitive to tetraethylammonium (TEA). A large fraction of KROS was inhibited by paxilline—a specific inhibitor of large-conductance Ca^2+^-activated BK channels. The TEA-insensitive component was inhibited by senicapoc—a specific inhibitor of the Ca^2+^-activated KCa3.1 channel. Both BK and KCa3.1 activation were mediated by an increase in [Ca^2+^]_i_ induced by Chl-T. Both KROS and [Ca^2+^]_i_ increase were inhibited by ACA and clotrimazole—two different inhibitors of the calcium-permeable TRPM2 channel. Surprisingly, IGR37 cells did not exhibit current increase upon the application of Chl-T. Expression analysis confirmed that the genes encoding BK, KCa3.1, and TRPM2 are much more expressed in IGR39 than in IGR37. The potassium currents and [Ca^2+^]_i_ increase observed in response to the oxidizing agent strongly suggest that these three molecular entities play a major role in the progression of melanoma. Pharmacological targeting of either of these ion channels could be a new strategy to reduce the metastatic potential of melanoma cells, and could complement classical radio- or chemotherapeutic treatments.

## 1. Introduction

Ion channels are specialized membrane proteins involved in several physiological functions, such as electrical signaling, signal transduction, and transport of ions small molecules across biological membranes in response to specific stimuli [1,2]. Nevertheless, ion channels can also be implicated in the development of a large variety of diseases where oxidative stress (OS) plays a major role [3], including neurodegenerative diseases [4], cardiovascular diseases [5], diabetes [6], aging [7], and cancer [8]. In this regard, the factors that favor the insurgence of cancer and its progression to a metastatic state depend on the production of free radicals, such as reactive oxygen species (ROS) [9,10]. In fact, the onset of many tumors occurs via the deleterious effects of free radicals associated with accumulation of damage over a period of years. Although the continuous accumulation of cellular and molecular changes is the major driving force for the initiation and progression of cancer, the underlying mechanisms can be promising targets for therapeutic mediation [8].

Oxidative stress is frequently described as the balance between the production of ROS in biological systems, and the ability of the latter to defend themselves through their endogenous antioxidant machinery [11,12,13]. Thus, when oxidants are produced in excess, or when the antioxidant defenses are ineffective, this balance can be perturbed, thus resulting in increased oxidative stress. In these conditions, biomolecules—such as nucleic acids, membrane lipids, enzymes, and proteins—can be damaged beyond their repair capacity. In this regard, ion channels possess sulfur-containing cysteine and methionine residues that can be targeted by ROS, thus altering channel function, including gating and conductivity properties, as well as associated signaling pathways [14]. Specifically, the amino acid methionine is particularly susceptible to oxidation due to the presence of a highly reactive sulfur atom within a hydrophobic side chain. In fact, in oxidative conditions induced by either endogenous species or exogenous reagents, methionine is oxidized to methionine sulfoxide (met-O), thus altering the secondary structure of the target protein [15]. Among oxidant agents, chloramine-T (Chl-T) efficiently oxidizes methionine, and represents an ideal tool to mimic abnormal intracellular ROS levels [16,17]. The chemical and physical changes caused by methionine oxidation drastically alter the functions of ion channels, and consequently, the effect of oxidative stress on ion channels could become an essential part of a potential pathogenic mechanism in cancer cells [16,18]. In fact, both the abnormal production of ROS and alterations in ion channel activity can promote many processes causing development and progression in a tumor cell, such as cellular proliferation, evasion of apoptosis or anoikis, metastasis, and angiogenesis [19,20].

Several types of K^+^ channels are overexpressed in cancer, and are believed to be important tumorigenic agents [21]. Nevertheless, due to the high number of ion channels and the high complexity of the field, progress in understanding is often slow and focused on single molecules. Specifically in melanoma, the role of ion channels has still not been elucidated in detail [22].

Malignant melanoma—a neoplasm arising from the malignant transformation of melanocytes—is predominantly a disease of the skin, but in rare instances can occur at other sites, including the mucous membranes and the eye [23]. It is one of the most aggressive and deadly skin cancers, and is surpassed only by lung cancer [24]. The vulnerability of melanocytes to oxidative stress can be explained by their great ability to produce melanin. In fact, the melanosome is thought to be the main source of the high levels of ROS observed in melanocytes and melanoma cells [20]. Therefore, a better molecular characterization is urgently needed in order to identify the key drivers and pathways involved. In addition, the identification of prognostic molecular markers of the metastatic process is critical for designing therapeutic modalities for reducing the occurrence of metastasis.

Among the ion channels expressed in melanoma, the large-conductance K^+^ channel—also known as the BK channel, and encoded by the *KCNMA1* gene—plays a complex and ambiguous role in human cancer [25,26,27,28,29]. BK channels are activated by voltage and intracellular calcium in a cooperative manner [30]. Expression of BK channels is increased in cancerous compared to healthy cells, and correlates with the malignancy of the tumors. It often contributes to the modulation of hormone-dependent cancers—such as breast, prostate, and cervical cancer—but it has also been proposed as a remedy target in other types of cancer, such as melanoma. Notably, it has been found that ROS modulate BK channel activity, although the effect appears to be variable, depending on the cell model used. For example, in fibroblasts, the oxidative stress inducer H_2_O_2_ favors BK channel activity via a mechanism involving PKC [31]. In HEK293 cells overexpressing BK channels, the effect of H_2_O_2_ was instead activatory or inhibitory, depending on the site of application—namely, a direct intracellular application, possibly involving cysteine oxidation, led to BK channel inhibition, whereas extracellular H_2_O_2_ application that activates the PI3K-dependent transduction pathway led to BK channel activation [32]. In addition, the extracellular application of oxidizing agents—such as 5′5-dithiobis(2-nitrobenzoic acid) (DTNB) or dithiothreitol (DTT)—significantly increases BK channel activity in the isolated smooth muscle cells of rabbit pulmonary arteries, resulting in a reduction in intracellular glutathione (GSH) levels, thus indicating that alterations by the GSH are connected with BK activity [33].

KCa3.1, encoded by the *KCNN4* gene, is another member of the Ca^2+^-activated K^+^ channel family, with a smaller single-channel conductance than BK, and lacking any significant voltage dependence. Its aberrant expression has been reported in several cancer types, such as colorectal and pancreatic cancers, where KCa3.1 channels are expressed on the inner mitochondrial membrane, and have been proposed to modulate important mitochondrial functions, such as oxidative phosphorylation [22,34,35,36,37].

Another class of ion channel implicated in cancer is transient receptor potential (TRP) channels. Since most TRP channels are permeable to Ca^2+^, increased TRP channel expression is expected to enhance intracellular Ca^2+^ levels and impact Ca^2+^ signaling. Indeed, overexpression of several TRP channel genes in various cancer types is compatible with a switch of cancer cells to an aberrant proliferative state [38]. Related to the aberrant proliferation and the often hypoxic environment, most cancers are characterized by enhanced oxidative stress [39,40], which can be directly sensed by various TRP channels, including TRPV1 [41], TRPV2 [17], and TRPM2 [42]. Interestingly, TRPM2 is implicated in several physiological and pathological pathways involving oxidative stress, and its activation has been generally associated with a large increase in intracellular Ca^2+^ [43,44,45,46,47,48].

Based on these considerations, the exploitation of ion channels as new molecular targets in cancer therapy [34,49,50,51,52] is a general goal. In the present investigation, the patch-clamp technique, quantitative measurements of intracellular Ca^2+^ levels, and RT-qPCR have been used to investigate the response to the oxidizing reagent Chl-T in IGR39 cells—a cell line derived from a human primary melanoma—and in IGR37 cells, which instead are derived from a metastasis of the same patient.

## 2. Results

### 2.1. Activation of Large Currents by Chl-T in IGR39 but Not in IGR37 Cells

**Figure 1:**
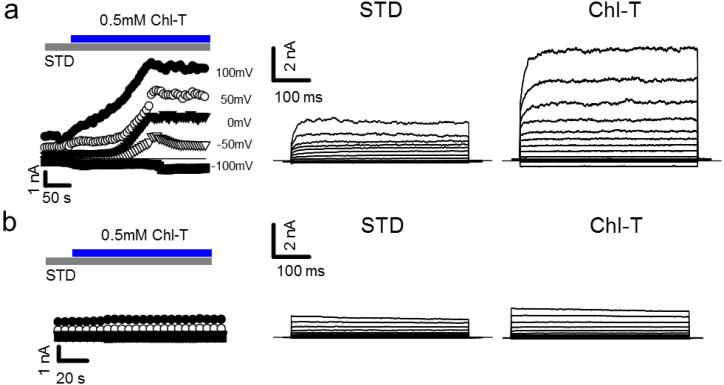
Application of Chl-T activates large outward currents in IGR39 cells, but not in IGR37 cells. (**a**) Left: time course of currents elicited in a typical IGR39 cell during the application of 0.5 mM Chl-T in STD solution, at different potentials from −100 mV to +100 mV, as indicated. Right: IV relationship in the same IGR39 cell elicited before and after the addition of Chl-T. (**b**) Similar to (**a**), but recorded in a representative IGR37 cell.

Tumor microenvironments are characterized by an increase in oxidative stress [39,40]. In order to investigate the electrophysiological responses of primary melanoma IGR39 cells to oxidative reagents in the whole-cell patch-clamp configuration, we applied the mild oxidant Chl-T. Invariably, perfusion of 0.5 mM Chl-T elicited very large outward currents, with a 3.3-fold increase at +100 mV compared to cells under standard conditions (normalized current increase at 100 mV: 3.29 ± 0.62, *p =* 0.0009, *n =* 16) (Figure 1a). After washout of Chl-T, currents slowly recovered, almost to the level preceding the application of the oxidant (data not shown). Surprisingly, application of Chl-T to IGR37 cells—metastatic cells derived from the same patient—did not elicit any current increase (normalized change of current at 100 mV: 0.86 ± 0.12, *p =* 0.266, *n =* 7) (Figure 1b).

### 2.2. Identification of the Large-Conductance (BK) K^+^ Channel as a Major Current Component Activated by Chl-T

**Figure 2:**
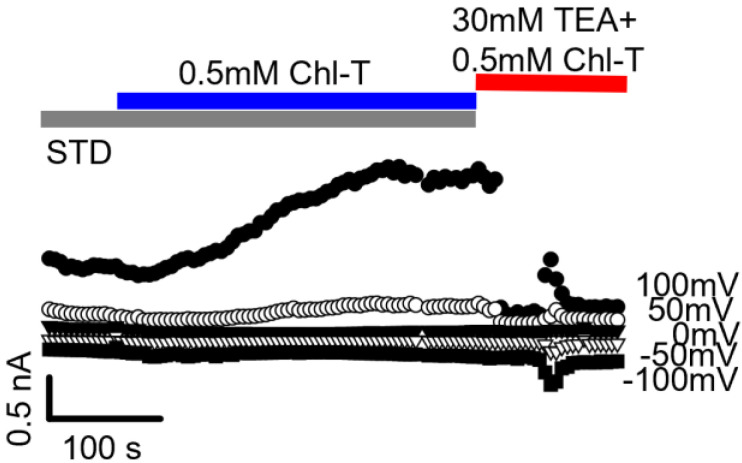
TEA blocks Chl-T-evoked outward currents. The figure shows a representative recording in which the addition of 30 mM TEA almost completely (~75%) inhibited currents elicited by the application of 0.5 mM Chl-T.

In order to investigate whether K^+^ channels are involved in the Chl-T-activated currents in IGR39 cells, we applied tetraethylammonium (TEA)—an inhibitor of several K^+^ channel classes (STD TEA, see Table 1). Figure 2 shows that the addition of 30 mM TEA almost completely inhibited Chl-T-evoked outward currents, confirming that they are mediated to a large extent by K^+^ currents, which we call KROS.

**Figure 3:**
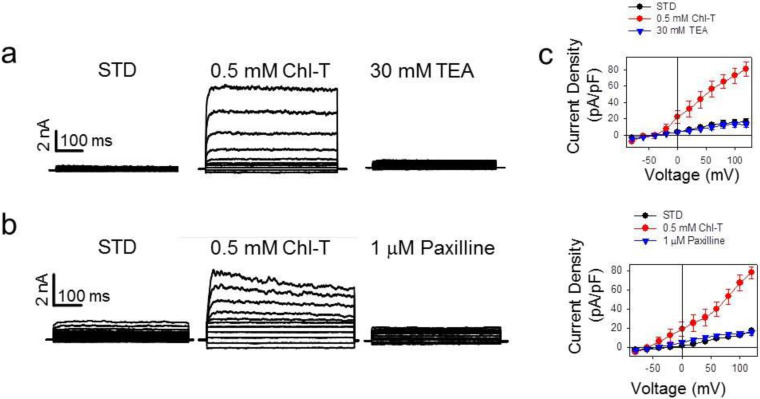
BK current activation by Chl-T oxidation in IGR39 cells. (**a**) Currents in a typical IGR39 cell elicited in STD solution (left) and after addition of 0.5 mM Chl-T (middle). Chl-T-evoked currents were blocked by 30 mM TEA (right traces). Average block at 100 mV: 81.9 ± 3.7% (*p =* 0.017, *n =* 8). (**b**) Representative currents recorded from another IGR39 cell. The potassium current, stimulated by oxidation, was blocked at positive voltages by 1 µM paxilline (right panel). Average block at 100 mV: 79.0 ± 2.5% (*p =* 0.0062, *n =* 6). (**c**) Average current density–voltage relationships for IGR39 cells treated with Chl-T and 30 mM TEA (top panel) and Chl-T and 1 µM paxilline (bottom). Data are expressed as mean ± SEM from 6 experiments each.

It is known that IGR39 cells express the large-conductance Ca^2+^-activated BK channel [53,54], and that these channels are activated by Chl-T [16]. In addition, BK channels are sensitive to the presence of TEA [55]. These results suggested that Chl-T-activated currents could be predominantly carried by BK channels. To confirm this hypothesis, we employed the specific BK inhibitor paxilline [56]. As observed for TEA, 1 µM paxilline also reduced the Chl-T-activated current by ~80% (Figure 3).

### 2.3. Additional TEA- and Paxilline-Insensitive K^+^ Current Activated by Chl-T

**Figure 4:**
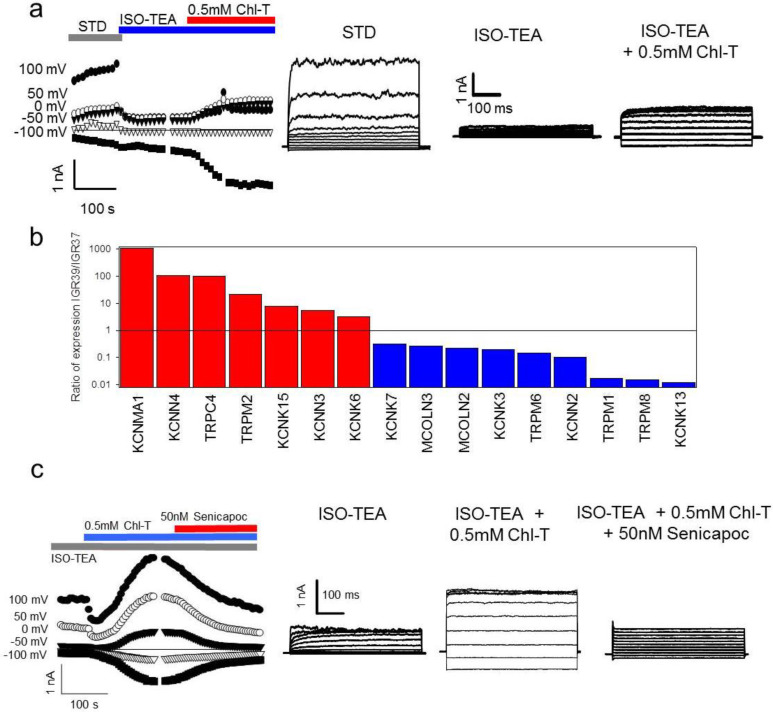
(**a**) Activation of TEA-insensitive current component by Chl-T. Right: time course of currents elicited in a representative IGR39 cell. Application of the ISO-TEA solution (blue bar) inhibited constitutively active BK-mediated currents. Subsequent addition of 0.5 mM Chl-T activated a significant current component. Left: IV relationships in the indicated conditions. (**b**) Comparative gene expression analysis of K^+^ and TRP ion channel genes in IGR39 vs. IGR37 cells. As expected, *KCNMA1* was highly expressed in IGR39 compared to IGR37 cells. Among other Ca^2+^-activated K^+^ channels, *KCNN4*-encoded KCa3.1 is more expressed in IGR39 cells, and among TRP channels, TRPC4 and TRPM2 are significantly more expressed in IGR39 cells. The analysis is based on the GSE137391 dataset, as described in the Materials and Methods section. The plot shows only genes with fold change > 2 (see also Table S2). The TRPA1 channel that has been reported to be activated by 0.1 mM Chl-T [64] is weakly expressed in both cell lines, and 1.6-fold more in IGR37 compared to IGR39 cells. (**c**) Inhibition of the TEA-insensitive component by senicapoc. Right: time course of currents elicited in a representative IGR39 cell kept initially in ISO-TEA, and then exposed to 0.5 mM Chl-T in ISO-TEA. Addition of 50 nM senicapoc—a specific inhibitor of KCa3.1—led to an immediate block of the TEA-insensitive potassium current. Left: IV relationships in the indicated conditions.

Closer inspection of KROS currents suggested the potential presence of a voltage-independent component (see, for example, the middle traces in Figure 3b), which appeared not to be completely inhibited by paxilline, and thus probably not mediated by BK. Therefore, we performed new experiments using an extracellular solution, in which the concentration of NaCl was further reduced to 60 mM in order to decrease any possible contamination by sodium conductances. To keep the osmolarity constant, NaCl was replaced with mannitol (ISO-TEA, see Table 1). Perfusion with ISO-TEA inhibited the initially dominant K^+^ conductance, most likely carried by BK channels (Figure 4a). Subsequent application of Chl-T activated significant inward and outward currents, with a reversal potential of about −55.5 ± 0.7 mV (*n =* 25)—relatively close to that of a current being predominantly carried by K^+^ (Figure 4a). The effect of paxilline on the current activated by Chl-T was less pronounced at negative potentials, supporting the hypothesis that Chl-T also stimulates K^+^ conductances insensitive to both TEA and paxilline, and thus different from BK channels. In fact, at −80 mV, paxilline inhibited Chl-T-evoked currents by only 28 ± 12% (*p =* 0.037, *n =* 6) (data not shown).

Several members of the K2P channel family are insensitive to TEA but sensitive to oxidizing conditions [57,58,59]. Thus, in order to determine which ion channel(s) underlie the observed current, we transfected TASK2, TREK1, TREK2, and TALK2 channels in HEK293 cells, and tested their sensitivity to Chl-T. However, none of these channels were significantly activated (Figure S2a–d).

Next, analysis of publicly available gene expression datasets of IGR39 and IGR37 cells revealed that the medium-conductance, Ca^2+^-activated, TEA-insensitive, voltage-independent channel gene *KCNN4* [60] is significantly more expressed in IGR39 than IGR37 cells (Figure 4b, Table S2). To test whether the *KCNN4*-encoded KCa3.1 channel underlies the TEA-insensitive component activated by Chl-T, we applied the specific KCa3.1 blocker senicapoc (bis(4-fluorophenyl)phenyl acetamide), which has a reported IC_50_ of 11 nM [61,62,63]. Figure 4c illustrates that 50 nM senicapoc clearly inhibited currents evoked by Chl-T in the presence of TEA. The reduction of the activated current was 65 ± 7% (*p =* 8.7 × 10^−5^, *n =* 4), demonstrating that the TEA-insensitive and voltage-independent components are indeed carried by KCa3.1 channels.

### 2.4. Intracellular Ca^2+^ Increase by the Application of Chl-T

**Figure 5:**
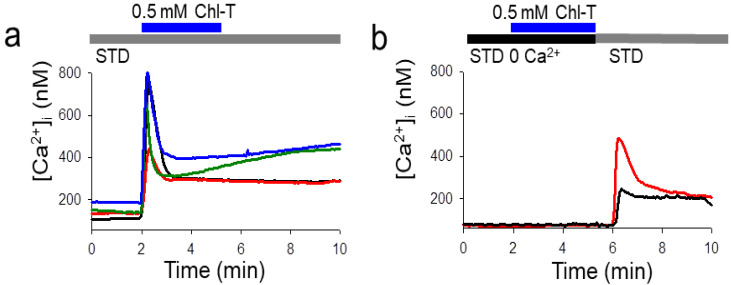
Application of Chl-T increases intracellular Ca^2+^ concentration. (**a**) Representative recordings of [Ca^2+^]_i_ in different IGR39 cells obtained from different cell preparations after the application of 0.5 mM Chl-T in the standard bath solution containing 2 mM Ca^2+^ (Δ[Ca^2+^]_i_ = 640 ± 30 nM (24 cells)). (**b**) Similar experiments obtained in the absence of Ca^2+^ in the external solution. Intracellular Ca^2+^ increase was observed only when Ca^2+^ was added to the bath solution. Horizontal bars indicate the time period of exposure. Note that in zero external calcium the resting cytosolic calcium is lower. Average resting values were 127 ± 9 nM in STD and 75 ± 2 nM without external calcium.

To test for possible mechanisms underlying the activation of both Ca^2+^-sensitive K^+^ channels (BK and KCa3.1), we employed fura-2 Ca^2+^ imaging. The application of Chl-T indeed induced a dramatic increase of [Ca^2+^]_i_ to ~800 nM (Figure 5a). The Ca^2+^ increase was completely dependent on the influx of extracellular Ca^2+^: [Ca^2+^]_i_ did not increase when a bath solution without added Ca^2+^ and with 3 mM EGTA (STD 0 Ca^2+^) was applied (Figure 5b), suggesting that Chl-T activates a plasma-membrane-localized Ca^2+^-permeable channel. No Ca^2+^ increase could be detected in IGR37 cells upon the application of Chl-T (Figure S1).

**Figure 6:**
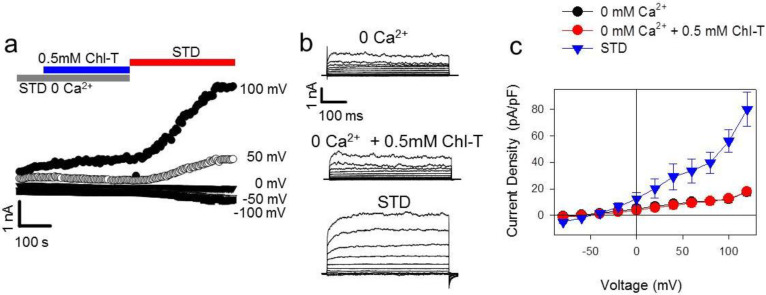
Application of Chl-T in the absence of extracellular Ca^2+^ does not activate K^+^ currents in IGR39 cells. (**a**) Time course of currents elicited in a representative IGR39 cell during the application of 0.5 mM Chl-T in 0-Ca^2+^ solution and subsequent application of STD solution. (**b**) Current traces before Chl-T application (left), during application in 0 Ca^2+^ (middle), and after perfusion with STD solution (right) (normalized Chl-T response in STD 0 Ca^2+^ solution at 100 mV = 1.08 ± 0.04, *p* = 0.7, *n* = 5; current fold increase after STD application 2.02 ± 0.22, *p* = 0.004, *n* = 4) (**c**) Current density–voltage relationship for IGR39 cells after the application of Chl-T in the absence of extracellular Ca^2+^, and subsequent STD administration. Data are expressed as mean ±SEM from 5 independent experiments. Vhold = −50 mV.

To further test the role of Ca^2+^ influx as the trigger of BK and KCa3.1 channel activation, we measured the current response to Chl-T in the absence of extracellular Ca^2+^. Under these conditions, the application of the oxidizing agent did not activate any significant current, until the extracellular solution was replaced by the Ca^2+^-containing STD solution (Figure 6).

This observation led us to hypothesize that Chl-T does not act directly on BK and KCa3.1 channels, but that it oxidizes and activates an unidentified Ca^2+^-permeable channel, whose activation induces an increase in intracellular Ca^2+^ and the consequent activation of the two Ca^2+^-activated potassium channels.

### 2.5. Identification of a Plasma Membrane Ca^2+^-Permeable Channel Sensitive to Oxidation

**Figure 7:**
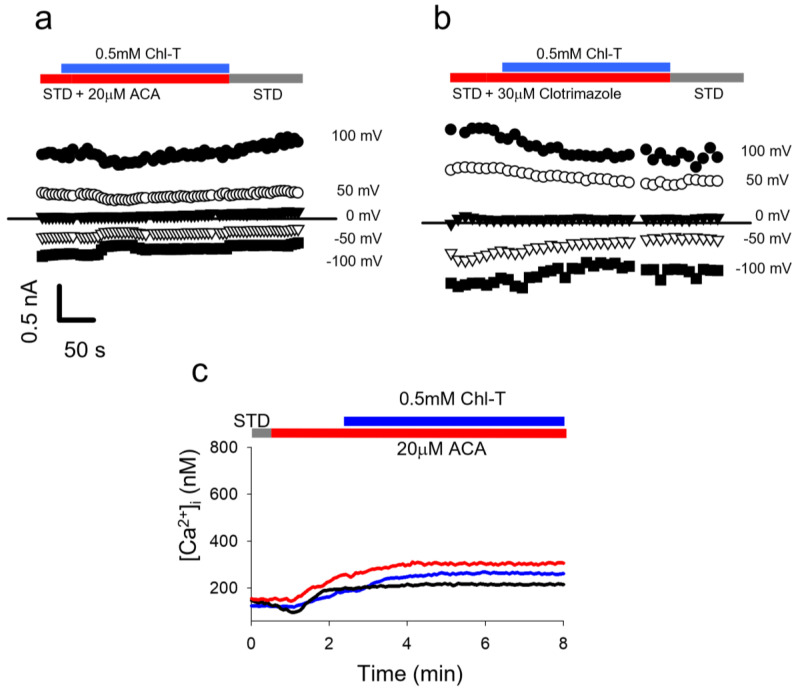
In IGR39 cells, TRPM2 blockers ACA and clotrimazole impede K^+^ current activation and [Ca^2+^]i increase by Chl-T. (**a**) Time course of currents recorded in a representative IGR39 cell during application in bath solution containing 0.5 mM ChlT and 20 µM ACA. (**b**) Similar to panel (**a**), but recorded during the addition of 30 µM clotrimazole. (**c**) Representative traces of [Ca^2+^]i recorded in IGR39 cells after the application of 0.5 mM Chl-T with 20 µM ACA in the standard bath solution containing 2 mM Ca^2+^.

From the gene expression analysis shown in Figure 4b, two Ca^2+^-permeable channels stand out as being more highly expressed in IGR39 vs. IGR37 cells: TRPC4 and TRPM2. We focused on TRPM2 because it is known to be activated by oxidation [65]. We performed Ca^2+^ imaging with fura-2-AM and patch-clamp experiments, and employed two TRMP2 inhibitors@ ACA (N-(p-amylcinnamoyl)anthranilic acid) and 1-(*o*-chloro-α,α-diphenylbenzyl)imidazole (clotrimazole) [65,66,67]. The administration of Chl-T in the presence of ACA or clotrimazole prevented the activation of K^+^ currents in whole-cell recordings (Figure 7a,b). Similarly, the presence of ACA almost completely impeded the Ca^2+^ influx in IGR39 cells induced by Chl-T ([Ca^2+^]_i_ increase: 15 ± 4 nM (*n =* 35), much smaller than in the absence of ACA, Figure 7c). Unfortunately, clotrimazole is able to release Ca^2+^ from the endoplasmic reticulum [68], making measurements using this inhibitor problematic (data not shown).

**Figure 8:**
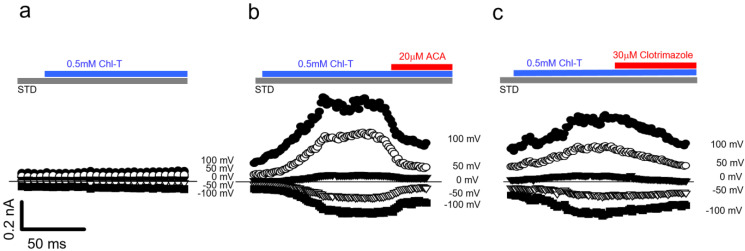
Activation of heterologously expressed TRMP2 by Chl-T. (**a**) Time course of currents elicited in an HEK-null cell during the addition of 0.5 mM ChlT in STD solution. (**b**,**c**) Time course of currents recorded in two representative transfected HEK293 cells during the application of 0.5 mM ChlT and subsequent addition of 20 µM ACA (**b**) or 30 µM clotrimazole (**c**). Normalized current increase at 100 mV: 2.18 ± 0.39, *p =* 0.007, *n =* 11. Average ACA block at 100 mV: 55.89 ± 1.92% (*p =* 0.00006, *n =* 4), and average clotrimazole block at 100 mV: 29.48 ± 1.97 (*p =* 0.0007, *n =* 4).

To further test the hypothesis that Chl-T activates TRPM2, leading to Ca^2+^ influx, we expressed human TRPM2 in HEK293 cells and performed Ca^2+^ imaging and patch-clamp experiments. The application of 0.5 mM Chl-T had no effect in non-transfected cells (Figure 8a), but induced a significant increase in currents in TRPM2-transfected cells (Figure 8b,c), which was reversed by 20 µM ACA (Figure 8b) or 30 µM clotrimazole (Figure 8c)—specific TRMP2 inhibitors. Thus, we conclude that Chl-T activates TRPM2, leading to Ca^2+^ influx and consequent activation of both BK and KCa3.1 channels.

### 2.6. Application of the Oxidizing Agent TBH70X Does Not Activate KROS

**Figure 9:**
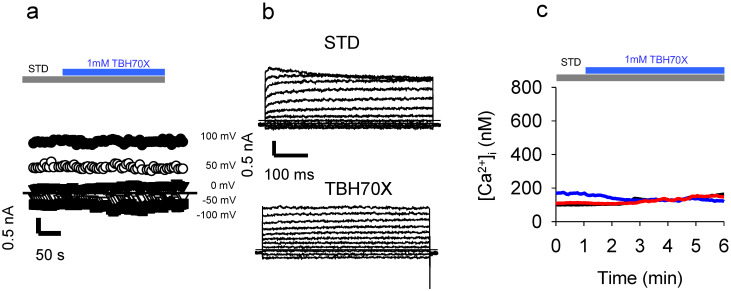
Application of TBH70X does not activate currents in IGR39 cells. (**a**) Time course of currents elicited in an IGR39 cell during the application of 1 mM TBH70X in STD solution, at different potentials from −100 mV to +100 mV, as indicated. (**b**) Representative current recorded in the same IGR39 cell elicited before and after the addition of 1 mM TBH70X. (**c**) Representative traces of [Ca^2+^]i after the application of 1mM TBH70X in the standard bath solution containing 2 mM Ca^2+^.

To test whether oxidation-mediated activation of KROS is specific to Chl-T, we applied TBH70X—another widely used oxidizing agent. Surprisingly, the application of 1 mM TBH70X to IGR39 cells did not elicit any current increase in three different experiments (Figure 9a,b). Similarly, TBH70X did not increase [Ca^2+^]_i_ (Figure 9b) ([Ca^2+^]_i_ increase: 13 ± 7 nM (*n =* 18)).

### 2.7. Channel Gene Expression Analysis

We performed quantitative real-time PCR experiments for a selected number of genes to confirm the gene expression data from the literature. In the first round, we screened all members of the family of K2P channels, which are voltage-independent and TEA-insensitive K^+^ channels and, thus, putative targets of the Chl-T response in IGR39 cells. Interestingly, one member of the family—*KCNK2* (TREK1)—showed higher expression in IGR39 than IGR37 cells (Figure S3). However, in absolute terms, TREK1 expression is relatively low in IGR39 cells. Furthermore, as shown in Figure S2, TREK1 is insensitive to Chl-T. Moreover, *KCNK7* was rather highly transcribed in both cell lines, consistent with data on human melanoma samples [69]. However, it is known from the literature that its expression does not give rise to any functional channel [70]. Consistent with this, we could not detect any channel activity in HEK cells transfected with a *KCNK7* plasmid, and Chl-T did not activate any K^+^ currents (data not shown).

**Figure 10:**
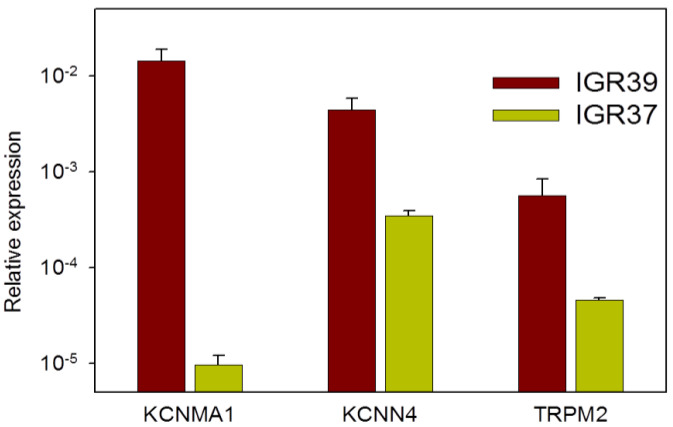
Expression of *KCNMA1*, *TRPM2*, and *KCNN4* in primary and metastatic melanoma cell lines. Expression is relative to that of the housekeeping genes actin or GAPDH (*n =* 2–3 experiments, each run in triplicate; see Materials and Methods).

Our qPCR analysis indicated that the expression of *KCNMA1*, encoding the alpha subunit of BK, was 1400-fold higher in IGR39 cells compared to their metastatic counterpart IGR37 (Figure 10), confirming the results from public datasets and from our functional analysis. *TRPM2* and *KCNN4* were also more expressed in IGR39 than IGR37 cells (Figure 10).

**Figure 11:**
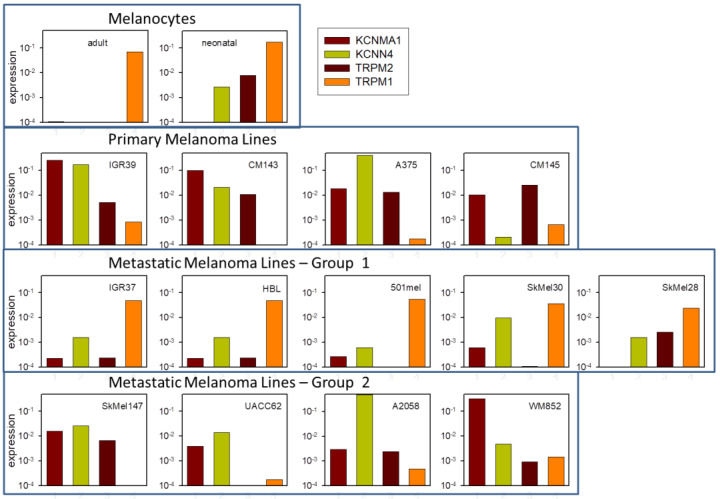
Expression analysis of selected ion channel genes in melanoma. Expression of the *KCNMA1*, *KCNN4*, *TRPM2*, and *TRPM1* genes in the indicated cell lines, as described in the Materials and Methods section.

In order to test whether the high expression and functionality of *KCNMA1*, *KCNN4*, and *TRPM2* is specific to (primary) IGR39 cells, we analyzed several publicly available datasets on gene expression in melanocytes, as well as primary and metastatic melanoma cell lines (Figure 11). Interestingly, while, as expected, melanocytes (adult and neonatal) showed little expression of these three genes, but high expression of TRPM1 (Figure 11, first row), all primary melanoma lines—including IGR39—showed a decreased expression of TRPM1 and highly increased expression of *KCNMA1* and *TRPM2*, and also of *KCNN4* in three out of four (Figure 11, second row). On the other hand, several metastatic lines—including IGR37—regained significant expression of TRPM1 and showed reduced expression of *KCNMA1*, *KCNN4*, and *TRPM2*, compared to the primary melanoma lines (Figure 11, third row). Other metastatic lines showed an intermediate expression pattern (Figure 11, fourth row).

## 3. Discussion and Conclusions

Oxidative stress reflects the imbalance between elevated ROS levels and the ability of the endogenous antioxidant system to readily detoxify the reactive intermediates or to repair cellular damage [71]. Most vulnerable to free radical attack is the cell membrane, as it contains a wide range of ion channels and other membrane proteins able to elicit different cellular reactions in response to extracellular stimuli and stressors. Therefore, the aim of the present study was to investigate the effects of the oxidizing reagent Chl-T on plasma membrane ion channel activity in primary (IGR39) and metastatic (IGR37) melanoma cells.

Based on a series of experiments, we could conclude that Chl-T activates the calcium-permeable TRPM2 channel, which is known to be sensitive to oxidation [42]. TRPM2 activation causes a dramatic increase in [Ca^2+^]_i_., which stimulates two distinct Ca^2+^-dependent K^+^ channels in IGR39 cells: the large-conductance voltage-dependent BK channel encoded by *KCNMA1*, and the medium-conductance voltage-independent KCa3.1 channel encoded by *KCNN4*.

To identify the elements involved in Chl-T-induced [Ca^2+^]_i_ increase and KROS activation—and, thus, to clarify the underlying molecular mechanism—we employed several pharmacological inhibitors: paxilline for BK channels, senicapoc for KCa3.1 channels, and clotrimazole and ACA for TRPM2 channels. Paxilline and senicapoc are rather specific for their targets among K^+^ channels [56,61,62,63]. Neither ACA nor clotrimazole are highly specific for TRPM2 [72]. For example, clotrimazole has been shown to activate the TRPA1 and TRPV1 channels [73]. Furthermore, ACA can act on several TRP channels, in the order TRPM2, TRPM8 > TRPC6 > TRPC3 [67,74,75]. Nevertheless, TRPM8, TRP6, and TRPC3 are little-expressed in IGR39 cells, and ACA does not appear to be effective on TRPC4 [76], which is instead highly expressed in IGR39 (see Figure 4b). In addition, the fact that both compounds—ACA and clotrimazole—inhibit Chl-T-induced KROS activation, and ACA blocks Chl-T-dependent [Ca^2+^]_i_ increase, strongly argues in favor of the hypothesis that oxidation of TRPM2 underlies the effects. Furthermore, the predominant role of TRPM2 was confirmed by showing that heterologously expressed TRPM2 was activated by Chl-T in HEK293 cells assayed by electrophysiology. Moreover, these conclusions were fully confirmed by PCR-based gene expression analysis of *KCNMA1*, *KCNN4*, and *TRPM2*, in perfect agreement with publicly available gene expression datasets (Figure 10 and Figure 11). Strictly, gene expression analysis does not prove that the respective proteins are expressed at an equivalent level. Complementary assays—for example, siRNA-mediated interference or Western blot—can provide additional evidence. For KCNMA1 and KCNN4, our functional and pharmacological evidence leaves little doubt of their strong expression in IGR39 cells, as also established by previous works [28,53,54]. In preliminary experiments, we tested the transfection of IGR39 cells via GFP-encoding plasmids, and found an extremely low transfection efficiency (data not shown). This renders the siRNA interference approach unfeasible. Furthermore, for TRPM2, no reliable antibodies are available. In any case, the combined evidence provided by electrophysiological, pharmacological, and gene expression analysis strongly suggests a significant expression of TRPM2 in IGR39 cells, and a weak expression in IGR37 cells.

It will be interesting to test whether the activation of TRPM2 is mediated by the oxidation of methionine 214 that has been identified as the target of H_2_O_2_-induced activation [77]. Conversely, the metastatic cell line IGR37, obtained from the same patient as the IGR39 cells, had a completely different behavior: no currents were activated upon Chl-T application, and no increase in intracellular Ca^2+^ levels could be detected (Figure S1). Again, the low expression of the three genes *KCNMA1*, *KCNN4*, and *TRPM2* is in agreement with these findings.

To test for the specificity of the action of Chl-T in IGR39 cells, we tested TBH70X—commonly used to model in vitro oxidant conditions—as it easily permeates the plasma membrane. Similarly to H_2_O_2_, TBH70X promotes OH^•^ radical formation via interaction with transition metals, usually present inside the cell, leading to subsequent cell injury [78]. Interestingly, TBH70X evoked neither BK channel activation nor an increase in [Ca^2+^]_i_ in IGR39 cells (Figure 9), confirming the specificity of Chl-T. Similarly, volume-regulated anion channels composed of LRRC8A and LRRC8C subunits are inhibited by Chl-T but not TBH70X [79], which could be explained by the hypothesis that methionines are the target of Chl-T, in that TBH70X is less efficient than Chl-T in oxidizing methionines compared—for example—to cysteines.

Interestingly, the application of Chl-T at elevated concentrations in inside-out patches leads to BK channel activation via a direct oxidation of intracellular methionines [16,80]. These findings are not consistent with our results in IGR39 cells, as Chl-T exposure in the absence of extracellular Ca^2^ did not impact BK currents in our experiments, thus indicating that a direct interaction between Chl-T and BK is unlikely to occur. The use of a low concentration and its application from the extracellular side are probably not sufficient to oxidize the intracellular methionines.

An important question is whether our conclusions are limited to IGR39 cells, or whether they could have more widespread relevance for other types of melanoma. The in silico gene expression analysis shown in Figure 11 strongly suggests that not only *KCNMA1* is highly expressed in primary melanoma cells, as already known [81], but also *KCNN4* and *TRPM2*. Conversely, several metastatic melanoma lines (including IGR37) neither express *KCNMA1* nor *KCNN4* or *TRPM2* in a significant manner, but regain a significant expression levels of TRPM1, while other metastatic lines are similar to primary melanoma (Figure 11). This observation is in line with the great heterogeneity of metastases [82].

The importance of oxidation-induced [Ca^2+^]_i_ increase and KROS activation may not be limited to melanoma. In proof-of-principle experiments, we investigated the effects of Chl-T in a primary tumor cell line from pancreatic duct cancer—namely, Panc-1. As illustrated in Figure S4a, stimulation of Panc-1 cells with 0.5 mM Chl-T evoked a large K^+^ current, with a 10-fold increase at 100 mV compared to the initial current. This Chl-T-induced current appeared to be very similar in terms of kinetics and voltage dependence to those we measured in Panc-1 cells when we employed the well-known BK activator NS-11021 (Figure S4b). Moreover, two independent RT-qPCR assays revealed that the BK channel is also expressed in this cell type (data not shown). The BK activation caused by Chl-T in Panc-1 cells is consistent with what Lin et al. demonstrated on the expression and role of TRPM2 in the proliferation and invasion of pancreatic ductal adenocarcinoma [83].

It is tempting to speculate that the *KCNMA1*, *KCNN4*, and *TRPM2* genes are functionally important for melanoma development, and can be pharmacological targets with metastatic potential. In fact, the BK channel has already been investigated as a target of cancer therapeutic drugs, although its role is very complex, and may not be universal [25,27,49,50,84,85,86,87]. Accumulating evidence has demonstrated that BK channels are overexpressed in some tumors compared with their healthy counterparts, thus playing a crucial role in controlling the cell cycle, cell proliferation, invasion, and metastasis [26,86,88]. In this regard, many of the pathophysiological phenotypes are associated with the upregulation of BK channels, confirming that the inhibition of BK channels might act as antitumor factor. In addition, the increased expression of *KCNMA1* in melanoma has been linked to the miRNA miR-211, which is encoded by intron 6 of *TRPM1*, and whose expression is inversely correlated with that of *KCNMA1* in primary melanoma cell lines and melanoma samples from patients [81]. It would be interesting to find out whether the expression of *KCNN4* and *TRPM2* is similarly linked to miR-211.

The oxidation sensitivity of TRPM2 is also particularly intriguing, and might be important in the tumor environment characterized by increased oxidative stress. Two basic cellular parameters could play a role in relation to these three ion channels: First, the dynamics of [Ca^2+^]_i_ influence myriad metabolic and signaling processes, including proliferation, gene expression, and migration. The activation of K^+^ channels tends to hyperpolarize the membrane potential, which can have direct, mostly inhibitory, effects on mitosis. In addition, a negative membrane potential increases the driving force for Ca^2+^ influx. In addition, KCa3.1 has been specifically involved in migration of melanoma cells [89].

In a similar manner, targeting KCa3.1 channels has been proposed as a strategy for pharmacological intervention in glioblastoma [90], in that the channel is also highly expressed and of likely importance in that tumor [91]. From a practical point of view, as highlighted by Brown et al. [90], senicapoc has already been tested in Phase III clinical trials, and would therefore be available for repurposing. It will be important in future research to uncover the specific functional roles of these ion channels in melanoma. Deciphering their roles will help in devising specific pharmacological tools targeting these three genes, aimed at interfering in melanoma expansion and metastasis. Nevertheless, it cannot be fully ruled out that the expression of the *KCNMA1*, *KCNN4*, and *TRPM2* genes in melanoma may be an epiphenomenon, i.e., unrelated to the tumor biology. However, even in this case, the widespread expression of these genes in primary and several metastatic melanomas could be considered to be an Achilles heel that can be exploited in drug treatment accompanying classical approaches. For example, activators of these channels could exacerbate the Ca^2+^ increase and promote apoptosis.

In conclusion, the high expression of the genes encoding for these ion channels in melanoma cells, together with their functional interrelation and concomitant activation by the oxidative conditions shown here, provides a promising route for pharmacological treatment. Specifically, targeting one or more of these channels could dramatically increase the effectiveness of classical treatments such as radio- or chemotherapy. This therapeutic strategy appears particular interesting, since melanoma is incurable when metastatic. Thus, this study could offer a good basis for the understanding of the relationship between oxidative stress and ion channels in melanoma. However, further investigations will be required in order to better clarify the involvement of these channels in tumorigenic events, such as cell viability, cell proliferation, and migration.

## 4. Materials and Methods

### 4.1. Cell Culture

The primary melanoma cell line IGR39, along with metastatic IGR37 cells, were obtained from Deutsche Sammlung von Mikroorganismen und Zellkulturen GmbH. They were derived from the same patient, and were cultured in DMEM medium, supplemented with 10% fetal calf serum, 2 mM L-glutamine, 100 µ/mL penicillin, 100 µg/mL streptomycin, 1% vitamin mix, and 1% non-essential amino acids. The HEK293 cell line was cultured in DMEM medium, supplemented with 10% fetal calf serum, 2 mM L-glutamine, 100 µ/mL penicillin, and 100 µg/mL streptomycin. All cell lines were maintained at 37 °C in a humidified 5% CO_2_/95% air atmosphere.

HEK293 cells were transiently transfected with Effectene^®^ Transfection Reagent (Qiagen), using 400 ng of a plasmid encoding the hTRPM2 channel, and 50 ng of a plasmid encoding CD8 [92]. Beads covered with anti-CD8 antibodies were used to identify positively transfected cells, as described in [92]. Currents were recorded 24–48 h after transfection.

### 4.2. Chemicals

All chemicals were purchased from Sigma. Stock solutions of paxilline, N-(p-amylcinnamoyl)anthranilic acid (ACA), bis(4-fluorophenyl)phenyl acetamide (senicapoc), and 1-(*o*-chloro-α,α-diphenylbenzyl)imidazole (clotrimazole) were prepared in DMSO and dissolved in the working solution immediately prior to the experiments. DMSO never exceeded 0.1%. Luperox^®^ TBH70X *tert*-Butyl hydroperoxide (TBH70X) solution, was dissolved in external working solution immediately prior to the experiments.

### 4.3. Patch-Clamp Experiments

For whole-cell patch-clamp experiments, the standard pipette (intracellular) solution contained (in mM): 8 NaCl, 100 K-Aspartate, 40 KCl, 10 EGTA, 10 HEPES, and 2 CaCl_2_ (pH 7.3). The free Ca^2+^ concentration in this solution was ~20 nM. The composition of the used extracellular solutions is indicated in Table 1.

Seal resistance was > 1 GΩ. The standard current–voltage protocol for stimulation consisted of 500-ms-long voltage steps from −80 to 120 mV, in 20 mV increments, starting from a holding potential of −50 mV. The response of patch-clamped cells to the various stimuli was monitored using the “time-course protocol”, which consisted of a “staircase” of 50-ms pulses to −100, −50, 0, 50, and 100 mV, administered every 5 s. No leak-current subtraction was performed. Membrane currents were filtered at 5 kHz and digitized at 20 kHz with a National Instruments DAQ interface employing the GePulse acquisition program (freely available at http://users.ge.ibf.cnr.it/pusch/programs-mik.htm), as described in [93,94,95]. Raw data were analyzed using the freely available Ana analysis program (http://users.ge.ibf.cnr.it/pusch/programs-mik.htm). Figures were prepared using SigmaPlot (Systat Software Inc. (SSI), San Jose, CA, USA).

### 4.4. Ca^2+^ Imaging with Fura-2-AM

Measurements of intracellular Ca^2+^ ([Ca^2+^]_i_) were performed using the fluorescent Ca^2+^ indicator fura-2 AM. Cells were loaded with 5 μM of fura-2 AM dissolved in extracellular solution with 0.1% pluronic acid to improve dye uptake, for 45 min at 37 °C. Cell coverslips were placed on the stage of an inverted Nikon TE200 fluorescence microscope (Nikon, Tokyo, Japan) equipped with a dual-excitation fluorometric Ca^2+^ imaging system (Hamamatsu, 325-6, Sunayama-cho, Naka-ku, Hamamatsu City, Shizuoka, Japan). Cells were excited at 340 and 380 nm, at a sampling rate of 0.5 Hz, and fluorescence emission, measured at 510 nm, was acquired with a digital CCD camera (Hamamatsu C4742-95-12ER). The external solutions were the same as used in the patch-clamp experiments. The fluorescence ratio F340/F380 was used to monitor [Ca^2+^]_i_ changes. Monochromator settings, chopper frequency, and data acquisition were controlled with dedicated software (Aquacosmos/Ratio U7501-01, Hamamatsu, Japan). [Ca^2+^]_i_ was calculated according to the method of Grynkiewicz et al. [96], using a KD of 140 nM for the Ca^2+^/fura-2 complex. Data were analyzed using SigmaPlot 8.0 software. Results are presented as mean ± standard error of different cells from at least 4 independent experiments.

### 4.5. Real-Time qPCR

Total RNA was extracted from IGR39 or IGR37 cells grown to subconfluency in a 25-cm^2^ flask, using the Aurum Total RNA Mini Kit (Bio-Rad, Hercules, CA, USA) according to the manufacturer’s instructions. RNA was quantified by measuring the absorbance with a spectrophotometer, and 1 µg was reverse-transcribed using the IScript advanced cDNA synthesis kit (Bio-Rad), including both oligo (dT) and random primers and the RNAse H+ MMLV reverse transcriptase enzyme. Alternatively, RNA was extracted with the PureLink RNA mini kit (Ambion) and 1 µg was reverse-transcribed using the SuperScript IV VILO cDNA synthesis kit (Thermo Fisher Scientific). The cDNAs obtained were used as templates for real-time PCR. Gene expression was generally assessed via SYBR Green quantitative real-time PCR using the SsoAdvanced Universal SYBR Green Supermix in the Bio-Rad CFX Connect Instrument. The thermal protocol consisted of a denaturation step at 95 °C for 3 min, followed by 39 two-step cycles composed of a denaturation step at 95 °C for 10 s and annealing/extension at 55 °C for 30 s. Amplification was performed using the oligonucleotide primers listed in Table S1. Primer design was performed with ProbeFinder (qpcr.probefinder.com, Roche) and checked for specificity with ensembl.org. When possible, primers were designed to span 2 exons, and amplicon length never exceeded 150 bp. The expression of each target gene was assessed in triplicate and normalized to the expression of the housekeeping gene actin. To avoid false positives, several controls—i.e., no template control (NTC), no primer control (NPC), and no reverse transcription control (NAC)—were included. At the end of amplification, a melting curve was run to check the specificity of the polymerization, and to visualize the presence of primer dimers or spurious products.

For the *KCNMA1* and *KCNK2* genes the expression was also verified with TAQMAN probes, using *GAPDH* as a reference housekeeping gene (Applied Biosystems Assay IDs: Hs02758991-g1 for GAPDH; Hs01119504_m1 for *KCNMA1*; Hs 01005159-m1 for *KCNK2*). The thermal protocol optimized for TaqMan gene expression consisted of an enzyme activation step at 95 °C for 2 min, followed by 39 two-step cycles, composed of denaturation for 3 s at 95 °C, and annealing/extension for 30 s at 60 °C. Results were visualized with Bio-Rad’s CFX manager software, while subsequent data analysis was performed using SigmaPlot (SPSS Inc., Chicago, IL, USA).

### 4.6. In Silico Gene Expression Analysis

The following GEO datasets were used for in silico analysis: GSE137391, GSE88741, GSE35704, and GSE107620. Expression data for each cell line and gene were normalized to the respective expression of the ribosomal *RPL19* gene. For Figure 4b, raw data from the GSE137391 dataset were aligned to the human genome. Sequences were trimmed using Trimgalore, and those that aligned to human rRNAs using Bowtie (v. 1.2.2 [97]) were discarded. Unmapped sequences were aligned to the human genome (GRCh38 Primary Assembly) using STAR (v.STAR-2.7.1a [98]) with Ensembl gene annotation (release 99). SAMtools (v. 1.8) was used to mark duplicated sequences, and StringTie (v. 1.3.6) was used to estimate gene abundances. EdgeR [99] was used for differential expression analysis, filtering genes with low expression (<5 counts). In Figure 8b, expression values for KCNMA1, KCNN4, TRPM2, and TRPM1 are shown for the melanocyte and melanoma cell lines of these datasets.

### 4.7. Statistics

Data are shown as representative experiments or as mean ± SEM. The statistical significance of differences between groups of data was assessed using Student’s *t*-test.

## Figures and Tables

**Table 1:** Composition of the extracellular solutions (in mM; pH was 7.3).

	NaCl	KCl	CaCl_2_	MgCl_2_	HEPES	TEACl	Mannitol	EGTA
STD	145	5	2	1	10	-	-	-
STD 0 Ca^2+^	145	5	-	1	10	-	-	3
STD TEA	115	5	2	1	10	30	-	-
ISO-TEA	60	5	2	1	10	30	100	-

## Data Availability

Original data are available from the authors on request.

## Supplementary Materials

The following are available online at https://www.mdpi.com/article/10.3390/ijms22168359/s1.
