# Supplementary material for: TRPM2 Oxidation Activates Two Distinct Potassium Channels in Melanoma Cells through Intracellular Calcium Increase
Source: Int J Mol Sci. 2021 Aug 4;22(16):8359. doi: 10.3390/ijms22168359 (PMC8393965; doi:10.3390/ijms22168359)
Supplement: Supplementary file 1 [file ijms-22-08359-s001.zip › ijms-1296681-supplementary.pdf]

Supplementary data

TRPM2 oxidation activates two distinct potassium channels in melanoma cells through intracellular calcium increase

Table S1. List of oligonucleotide primers used in real time qPCR. The primers were designed as indicated in the methods section.

Table S1. Primers used in real time qPCR

Gene	*Forward*	*Reverse*
KCNK1	GCTCTTCTTCGCCAGCAC	CTGACAAGGGCACGGTGT
KCNK2	TGCCAAAGTGGAAGATACGTT	CAGGCAGAGCCACAAAGAG
KCNK3	CCTTCTACTTCGCCATCACC	GAACATGCAGAACACCTTGC
KCNK4	TGGGCTTTGGCGACTATG	AGCACTGAGGCGAAGTAAGC
KCNK5	GAGGGCCTCTACTACTCCTTCAT	GTACAGGGCGTGGTAGTTGG
KCNK6	GTCGCACCAGCAACTCTCT	AGCTACCTGGGGATGGAAG
KCNK7	CCTGGGACCTTCCCTCAG	CGATAGTGGGGCCATGTG
KCNK9	TCCTTCTACTTTGCGATCACG	ACGGCGTAGAACATGCAGA
KCNK10	AGTCCAATAGGAAACTCTTCCAAC	CGGAGCAATATTCCCATACC
KCNK12	GCATTTACTCGCTCTTCAACG	GCAGCATCCAGTTGAGCAC
KCNK13	GGCGCCTTCTACTTCGTG	TACTGTCGCCGGAGTTGTC
KCNK15	TAGAATGACGGGGTTCATCC	GAAAGTCCTGCCAGAGATGC
KCNK16	AGGCACAGTCGTCACTACCA	CCAACAGGGCATAGAAGACAC
KCNK17	CAGCAACACCACCAGCAT	GCTCAGGTTGCCATAGCC
KCNK18	GCAGCAGATGATGGAGAGTTT	CCTGTTTTCTGTCTTCCACCA
KCNN4	GCTGCGTCTCTACCTGGTG	CGATGCTGCGGTAGGAAG
TRPM2	CGAGCTGGAGAGAGGACTGT	AGGGCTCCATTCTGAGACC
ACTB	CTGGAACGGTGAAGGTGACA	AAGGGACTTCCTGTAACAAT

Table S2. Differentially expressed genes between IGR39 and IGR37 cell lines. Data from GEO dataset GSE137391 were aligned to human genome as described in main text. EdgeR was used to obtain fold-change in expression, p-values and Benjamini-Hochberg adjusted p-value. We reported genes with more than 5 counts in at least one sample. Using a threshold adj.p-value < 0.05, KCNMA1, KCNN4 and TRPC4 result significantly upregulated in IGR39 cells compared to IGR37 while TRPM1, TRPM8, KCNK13 are downregulated in the same cells.

Table S2. Differentially expressed genes in dataset GSE137391

Gene	Ratio IGR39/IGR37	P-value	Adjusted P-Value
**KCNMA1**	256.47	3E-5	0.02
**TRPM1**	0.02	8E-5	0.02
**TRPM8**	5E-3	5E-4	0.02
**KCNN4**	19.31	8E-4	0.03
**KCNK13**	8E-3	1E-3	0.03
**TRPC4**	101.45	2E-3	0.04
**KCNK5**	0.01	3E-3	0.05
**TRPM2**	8.11	0.01	0.10
**MCOLN2**	0.29	0.02	0.14
**KCNN3**	35.13	0.02	0.14
**KCNN2**	0.09	0.03	0.20
**KCNK6**	3.85	0.04	0.21
**TRPM7**	0.52	0.06	0.28
**MCOLN3**	0.40	0.08	0.34
**TRPV1**	5.36	0.09	0.36
**KCNK2**	2.66	0.11	0.40
**TRPC1**	1.94	0.19	0.53
**TRPM4**	1.82	0.26	0.62
**TRPV2**	0.83	0.38	0.73
**MCOLN1**	0.72	0.48	0.79
**KCNK15**	0.68	0.59	0.85
**TRPM3**	0.82	0.79	0.94
**KCNN1**	0.95	0.93	0.98

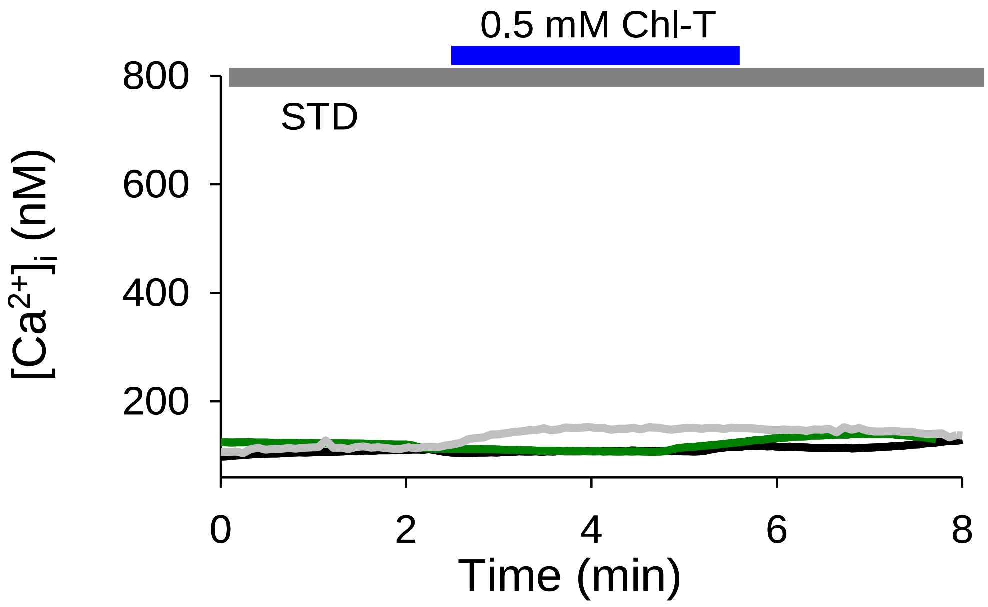

Figure S1. In IGR37, application of Chl-T does not increase intracellular Ca^{2+} concentration. Representative recordings of $[Ca^{2+}]_i$ in different IGR37 cells obtained from different cells preparations after the application of 0.5 mM Chl-T in the standard bath solution containing 2 mM Ca^{2+}

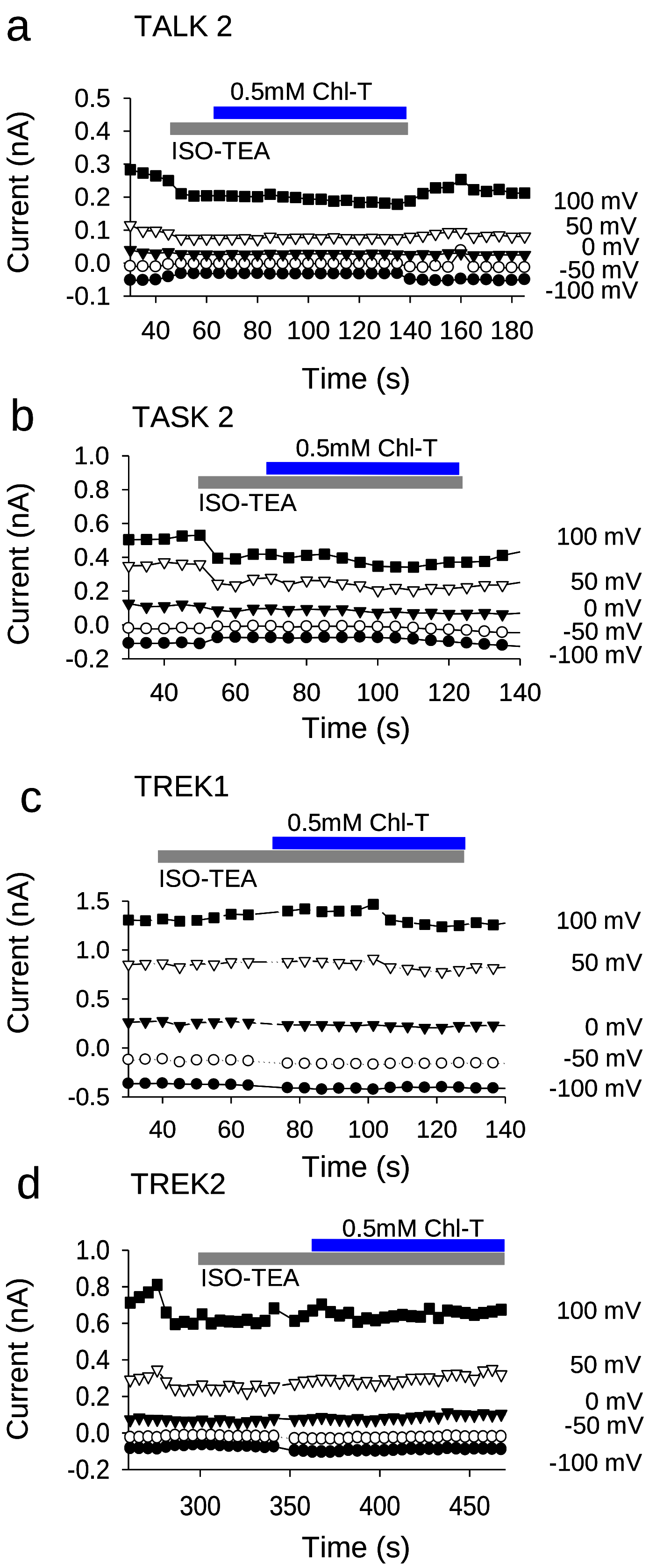

Figure S2. Candidate potassium channels. K2P channels, namely TALK2 (KCNK17) (a), TASK2 (KCNK5) (b), TREK1 (KCNK2) (c) and TREK2 (KCNK10) (d) were separately transfected in HEK 293 cells and tested for their sensitivity to Chl-T oxidation. None of them showed any response. The HEK-293 cell line was cultured in DMEM medium, supplemented with 10% fetal calf serum, 2 mM L-glutamine, 100 U/ml penicillin, 100 µg/ml streptomycin. Cells were maintained at 37 °C in a 5% CO_2 / 95% air atmosphere. Cells

were transfected by means of the Effectene Kit from Qiagen, using 400 ng of each plasmid expressing different K2P channels and 50 ng of a vector expressing CD8. Currents were recorded 24–48 h after transfection incubating cells at 37°C.

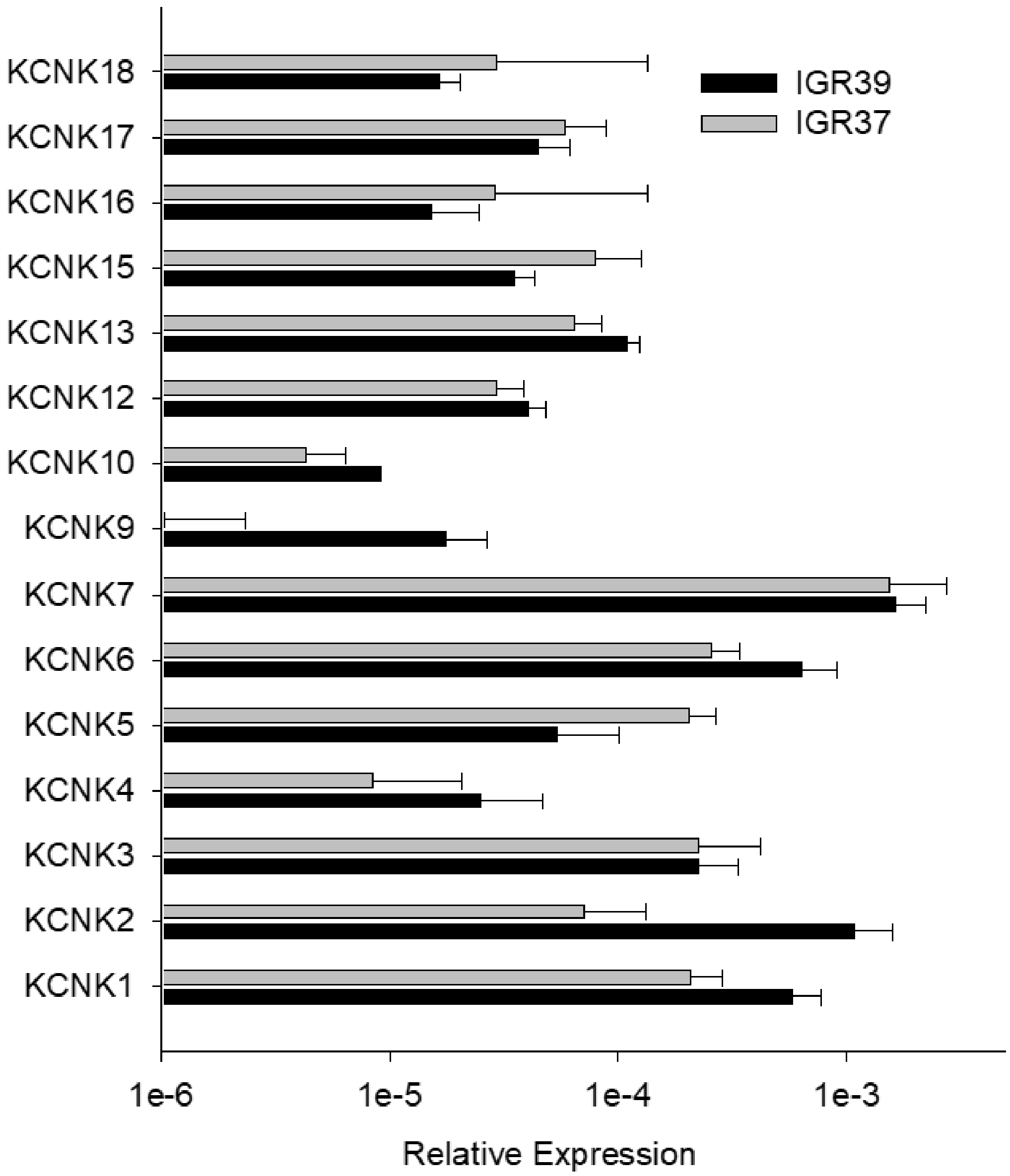

Figure S3. Expression profile of K2P channels. Expression is relative to that of the house-keeping genes Actin or GAPDH (n=2-3 experiments, each one run in triplicate; see Methods).

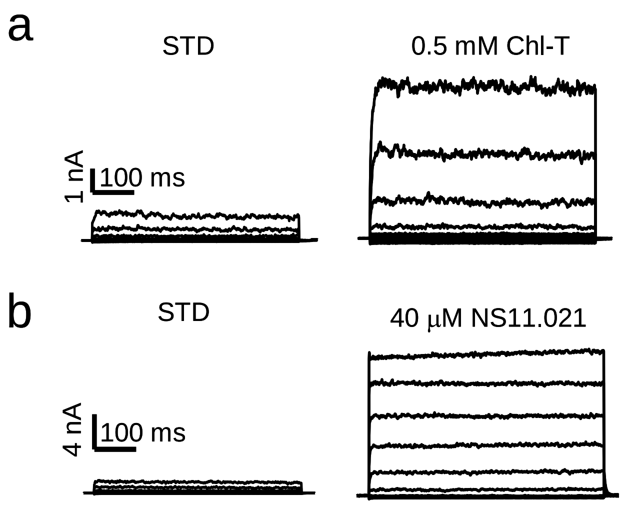

Figure S4. Activation of Potassium currents in Panc-1 cell line. a. Activation of large potassium current by 0.5 mM Chl-T. b. BK currents evoked by the specific BK activator NS11021 (40 M)
